# Dr. Corydon A. Woodward

**Published:** 1903-08

**Authors:** 


### Obituary.

### DR. CORYDON A. WOODWARD.

On the 7th of March, 1903, there passed from among us through the gate of death one of our most beloved and respected members,—Dr. Corydon A. Woodward.

Born in the State of Maine sixty-three years ago, he studied dentistry in the Baltimore Dental College, from whence he was graduated with credit, and for a time practised his profession in Rhode Island.

Removing to Cuba, he lived there eight years, but the unsettled condition of affairs there at that time compelled him to return to this country.

In 1871 he came to New York and became Professor of Prosthetics in the New York College of Dentistry.

He soon became known as a skilful and honorable practitioner, and took a high rank in the profession.

He was president of the New York Odontological Society in 1892–93. He assisted in the organization of The New York Institute of Stomatology, the first meeting of which was held at his house, April 19, 1895, and became its vice-president. Always active and zealous in promoting its object and interests, he gave to it the benefit of his sound judgment and advice, proving himself

"A friend of truth, of soul sincere,
In action faithful and in honor clear."

In recognition of his virtue and our source of loss in his death, we ask that this brief testimonial be placed upon the records of this society.

A. H. BROCKWAY,
GEO. S. ALLAN,
S. E. DAVENPORT,
*Committee The New York Institute of Stomatology.*

